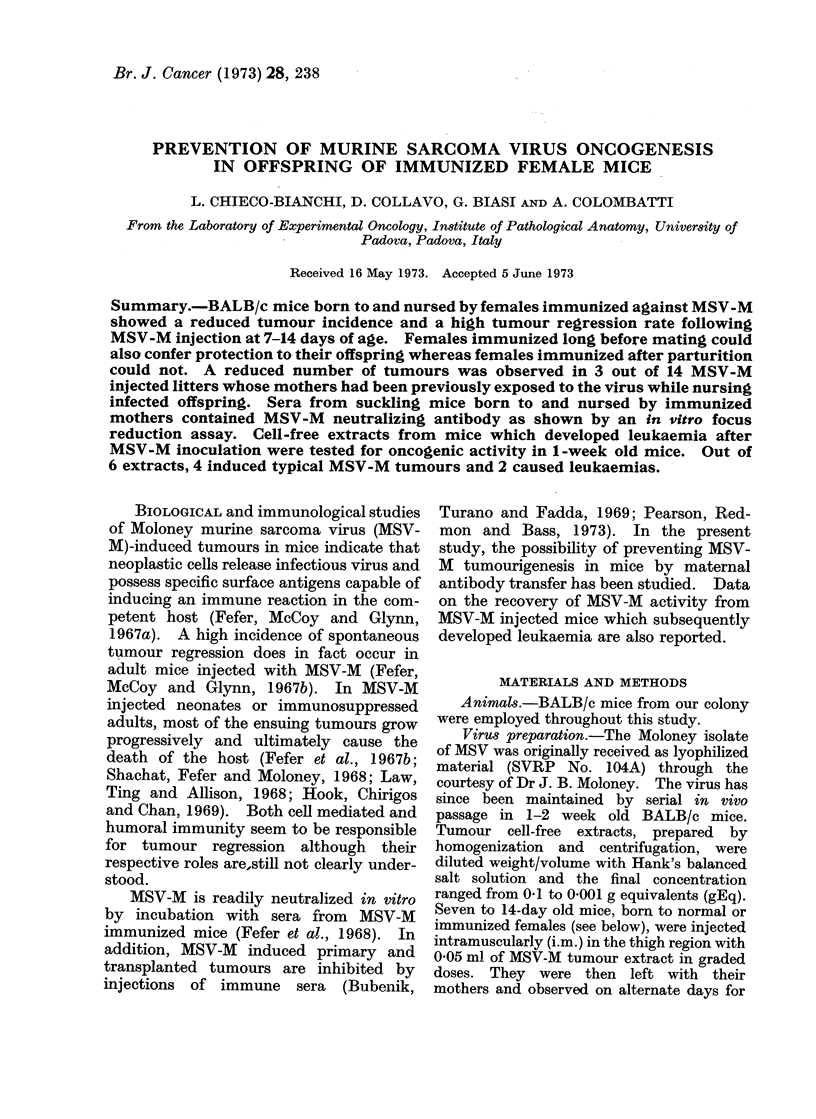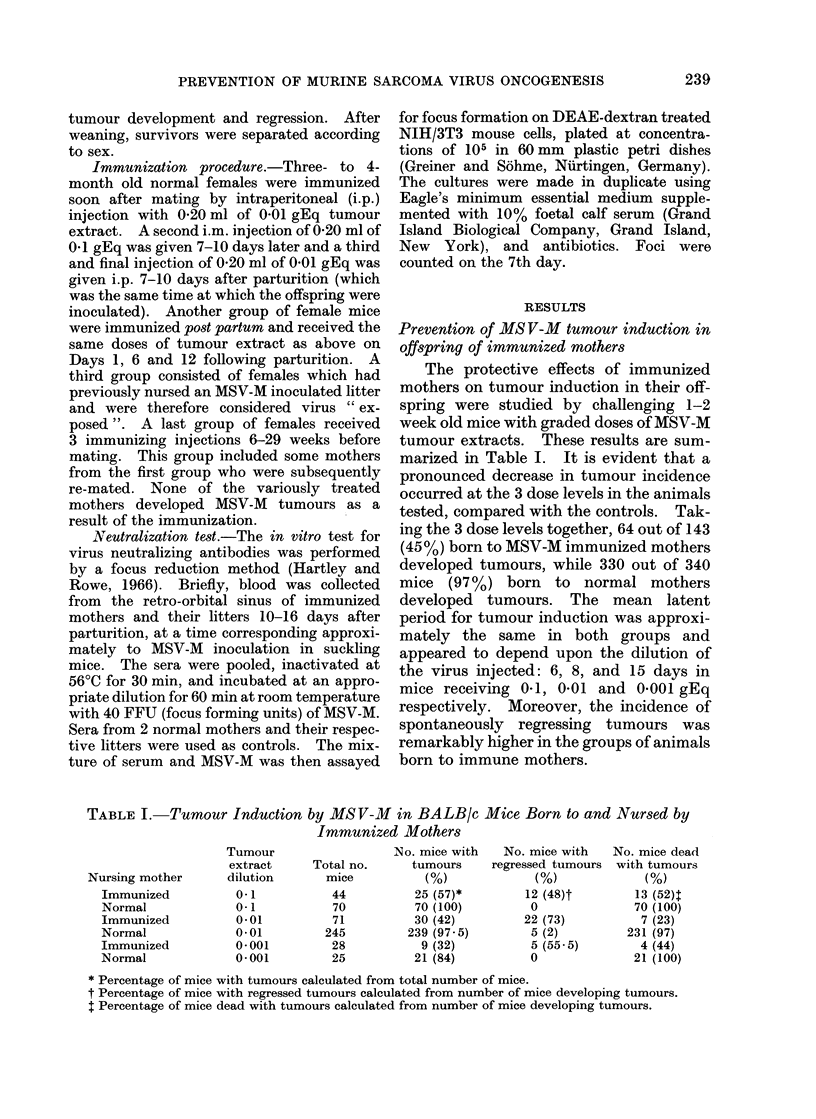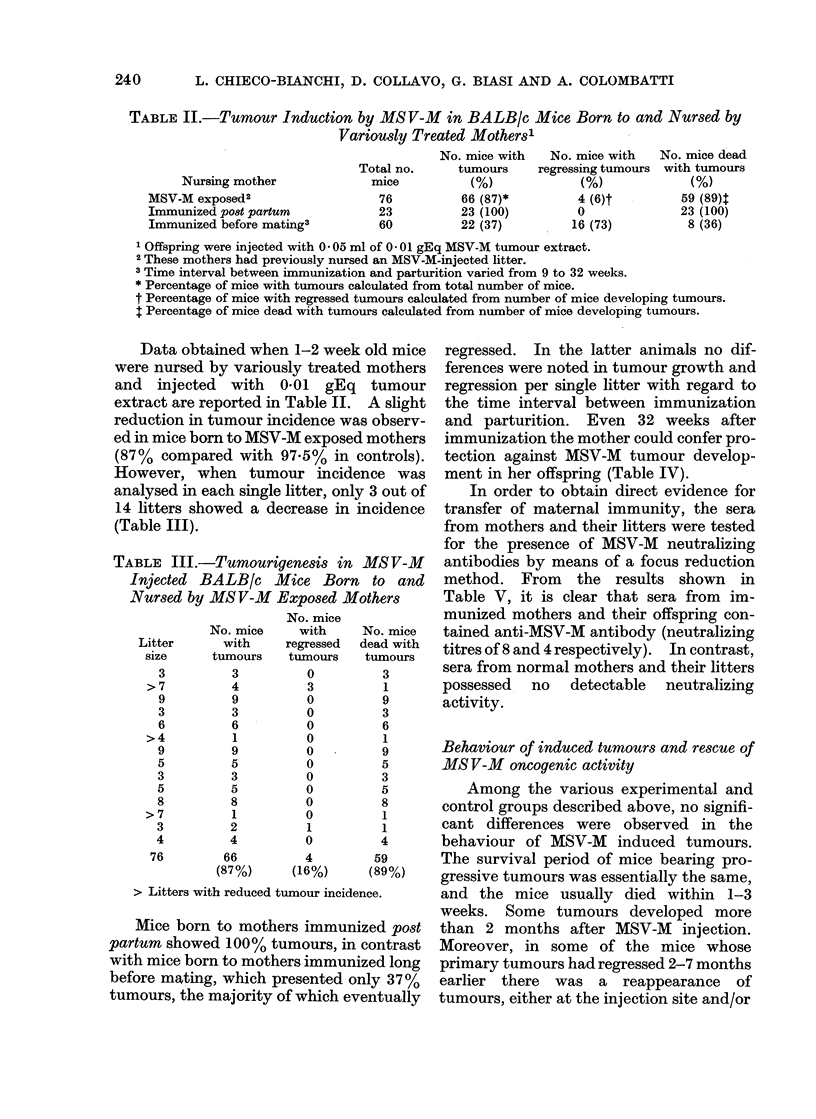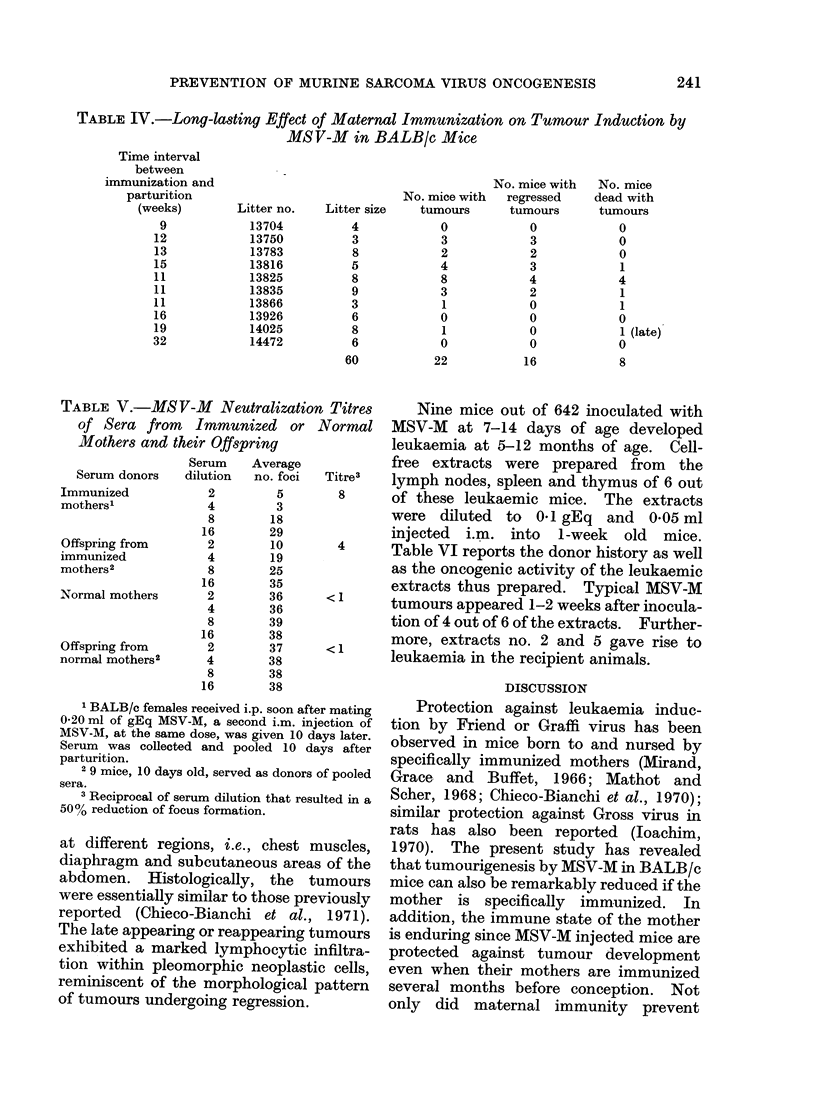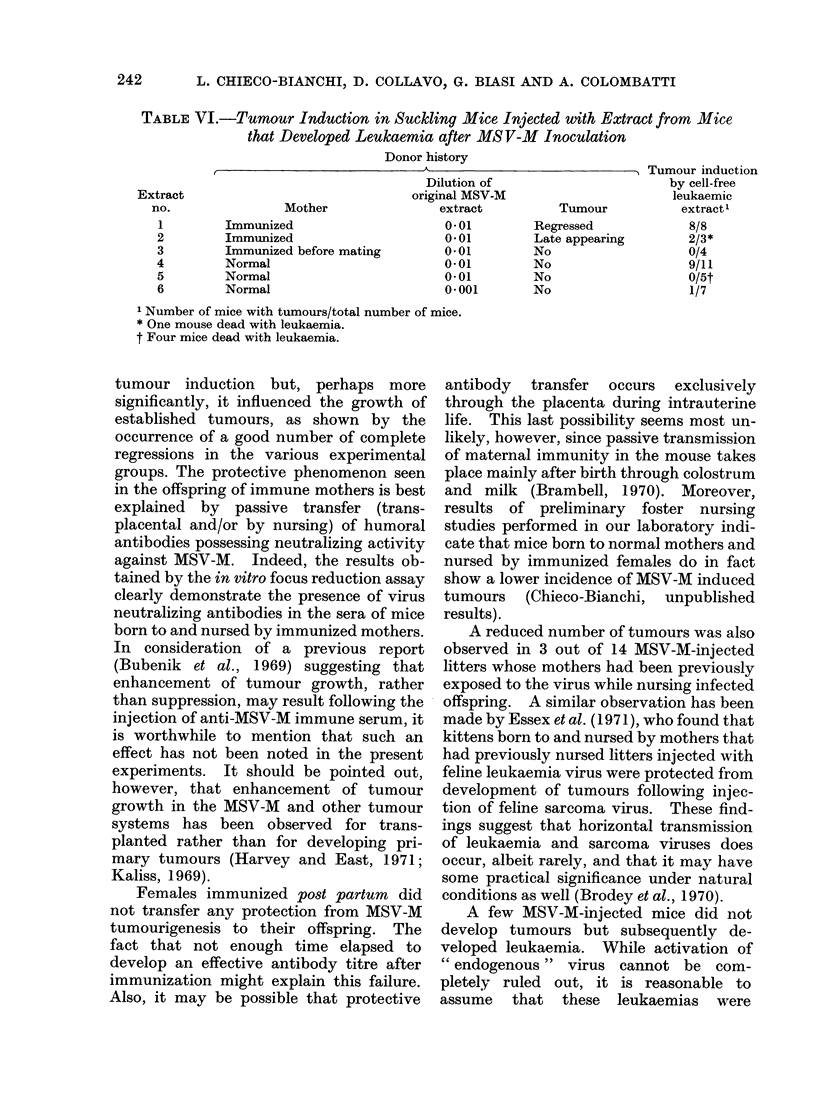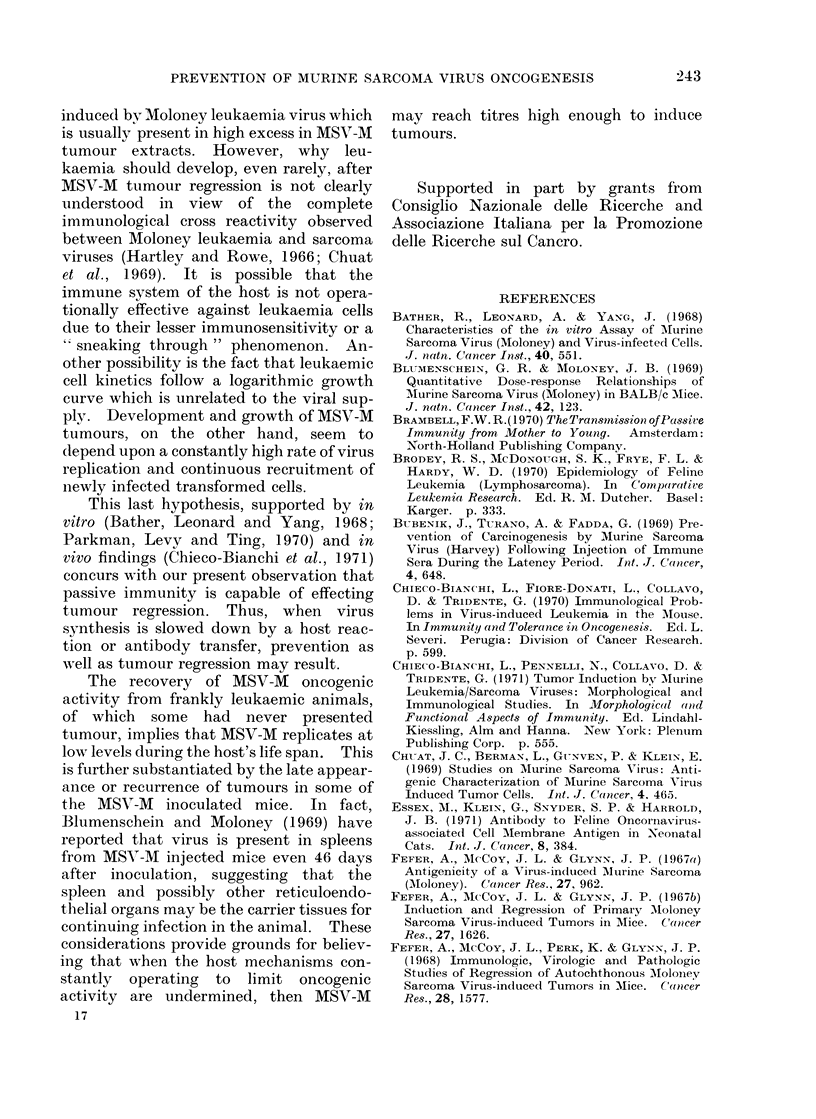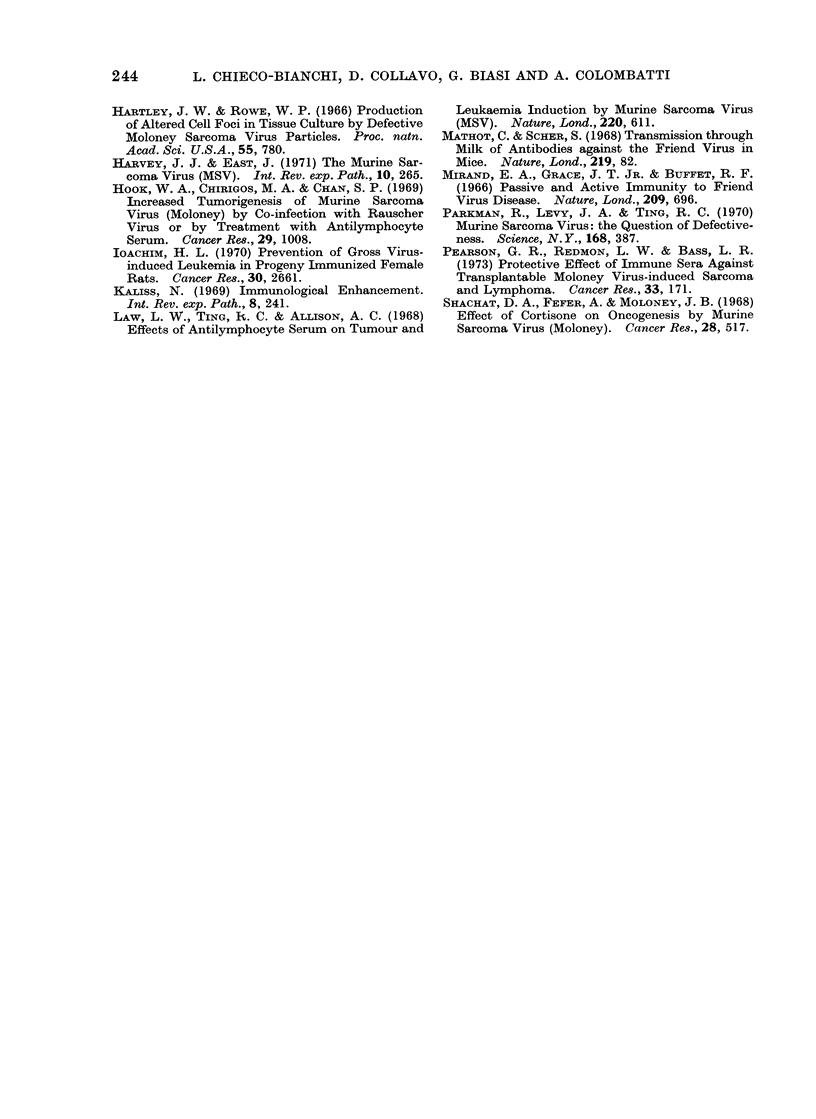# Prevention of Murine Sarcoma Virus Oncogenesis in Offspring of Immunized Female Mice

**DOI:** 10.1038/bjc.1973.143

**Published:** 1973-09

**Authors:** L. Chieco-Bianchi, D. Collavo, G. Biasi, A. Colombatti

## Abstract

BALB/c mice born to and nursed by females immunized against MSV-M showed a reduced tumour incidence and a high tumour regression rate following MSV-M injection at 7-14 days of age. Females immunized long before mating could also confer protection to their offspring whereas females immunized after parturition could not. A reduced number of tumours was observed in 3 out of 14 MSV-M injected litters whose mothers had been previously exposed to the virus while nursing infected offspring. Sera from suckling mice born to and nursed by immunized mothers contained MSV-M neutralizing antibody as shown by an *in vitro* focus reduction assay. Cell-free extracts from mice which developed leukaemia after MSV-M inoculation were tested for oncogenic activity in 1-week old mice. Out of 6 extracts, 4 induced typical MSV-M tumours and 2 caused leukaemias.


*Br. J. Cancer* (1973) **28**, 238

### PREVENTION OF MURINE SARCOMA VIRUS ONCOGENESIS IN OFFSPRING OF IMMUNIZED FEMALE MICE

L. CHIECO-BIANCHI, D. COLLAVO, G. BIASI AND A. COLOMBATTI

*From the Laboratory of Experimental Oncology, Institute of Pathological Anatomy, University of Padova, Padova, Italy*

Received 16 May 1973. Accepted 5 June 1973

**Summary.—BALB/c mice born to and nursed by females immunized against MSV-M showed a reduced tumour incidence and a high tumour regression rate following MSV-M injection at 7–14 days of age. Females immunized long before mating could also confer protection to their offspring whereas females immunized after parturition could not. A reduced number of tumours was observed in 3 out of 14 MSV-M injected litters whose mothers had been previously exposed to the virus while nursing infected offspring. Sera from suckling mice born to and nursed by immunized mothers contained MSV-M neutralizing antibody as shown by an *in vitro* focus reduction assay. Cell-free extracts from mice which developed leukaemia after MSV-M inoculation were tested for oncogenic activity in 1-week old mice. Out of 6 extracts, 4 induced typical MSV-M tumours and 2 caused leukaemias.**

BIOLOGICAL and immunological studies of Moloney murine sarcoma virus (MSV-M)-induced tumours in mice indicate that neoplastic cells release infectious virus and possess specific surface antigens capable of inducing an immune reaction in the competent host (Fefer, McCoy and Glynn, 1967*a*). A high incidence of spontaneous tumour regression does in fact occur in adult mice injected with MSV-M (Fefer, McCoy and Glynn, 1967*b*). In MSV-M injected neonates or immunosuppressed adults, most of the ensuing tumours grow progressively and ultimately cause the death of the host (Fefer *et al.*, 1967*b*; Shachat, Fefer and Moloney, 1968; Law, Ting and Allison, 1968; Hook, Chirigos and Chan, 1969). Both cell mediated and humoral immunity seem to be responsible for tumour regression although their respective roles are still not clearly understood.

MSV-M is readily neutralized *in vitro* by incubation with sera from MSV-M immunized mice (Fefer *et al.*, 1968). In addition, MSV-M induced primary and transplanted tumours are inhibited by injections of immune sera (Bubenik, Turano and Fadda, 1969; Pearson, Redmon and Bass, 1973). In the present study, the possibility of preventing MSV-M tumourigenesis in mice by maternal antibody transfer has been studied. Data on the recovery of MSV-M activity from MSV-M injected mice which subsequently developed leukaemia are also reported.

### MATERIALS AND METHODS

*Animals.*—BALB/c mice from our colony were employed throughout this study.

*Virus preparation.*—The Moloney isolate of MSV was originally received as lyophilized material (SVRP No. 104A) through the courtesy of Dr J. B. Moloney. The virus has since been maintained by serial *in vivo* passage in 1–2 week old BALB/c mice. Tumour cell-free extracts, prepared by homogenization and centrifugation, were diluted weight/volume with Hank's balanced salt solution and the final concentration ranged from 0·1 to 0·001 g equivalents (gEq). Seven to 14-day old mice, born to normal or immunized females (see below), were injected intramuscularly (i.m.) in the thigh region with 0·05 ml of MSV-M tumour extract in graded doses. They were then left with their mothers and observed on alternate days for tumour development and regression. After weaning, survivors were separated according to sex.

*Immunization procedure.*—Three- to 4-month old normal females were immunized soon after mating by intraperitoneal (i.p.) injection with 0·20 ml of 0·01 gEq tumour extract. A second i.m. injection of 0·20 ml of 0·1 gEq was given 7–10 days later and a third and final injection of 0·20 ml of 0·01 gEq was given i.p. 7–10 days after parturition (which was the same time at which the offspring were inoculated). Another group of female mice were immunized *post partum* and received the same doses of tumour extract as above on Days 1, 6 and 12 following parturition. A third group consisted of females which had previously nursed an MSV-M inoculated litter and were therefore considered virus " exposed ". A last group of females received 3 immunizing injections 6–29 weeks before mating. This group included some mothers from the first group who were subsequently re-mated. None of the variously treated mothers developed MSV-M tumours as a result of the immunization.

*Neutralization test.*—The *in vitro* test for virus neutralizing antibodies was performed by a focus reduction method (Hartley and Rowe, 1966). Briefly, blood was collected from the retro-orbital sinus of immunized mothers and their litters 10–16 days after parturition, at a time corresponding approximately to MSV-M inoculation in suckling mice. The sera were pooled, inactivated at 56°C for 30 min, and incubated at an appropriate dilution for 60 min at room temperature with 40 FFU (focus forming units) of MSV-M. Sera from 2 normal mothers and their respective litters were used as controls. The mixture of serum and MSV-M was then assayed for focus formation on DEAE-dextran treated NIH/3T3 mouse cells, plated at concentrations of $10^5$ in 60 mm plastic petri dishes (Greiner and Söhme, Nürtingen, Germany). The cultures were made in duplicate using Eagle's minimum essential medium supplemented with 10% foetal calf serum (Grand Island Biological Company, Grand Island, New York), and antibiotics. Foci were counted on the 7th day.

### RESULTS

### *Prevention of MSV-M tumour induction in offspring of immunized mothers*

The protective effects of immunized mothers on tumour induction in their offspring were studied by challenging 1–2 week old mice with graded doses of MSV-M tumour extracts. These results are summarized in Table I. It is evident that a pronounced decrease in tumour incidence occurred at the 3 dose levels in the animals tested, compared with the controls. Taking the 3 dose levels together, 64 out of 143 (45%) born to MSV-M immunized mothers developed tumours, while 330 out of 340 mice (97%) born to normal mothers developed tumours. The mean latent period for tumour induction was approximately the same in both groups and appeared to depend upon the dilution of the virus injected: 6, 8, and 15 days in mice receiving 0·1, 0·01 and 0·001 gEq respectively. Moreover, the incidence of spontaneously regressing tumours was remarkably higher in the groups of animals born to immune mothers.

TABLE I.—*Tumour Induction by MSV-M in BALB/c Mice Born to and Nursed by Immunized Mothers*

| Nursing mother | Tumour extract dilution | Total no. mice | No. mice with tumours (%) | No. mice with regressed tumours (%) | No. mice dead with tumours (%) |
|---|---|---|---|---|---|
| Immunized | 0·1 | 44 | 25 (57)* | 12 (48)† | 13 (52)‡ |
| Normal | 0·1 | 70 | 70 (100) | 0 | 70 (100) |
| Immunized | 0·01 | 71 | 30 (42) | 22 (73) | 7 (23) |
| Normal | 0·01 | 245 | 239 (97·5) | 5 (2) | 231 (97) |
| Immunized | 0·001 | 28 | 9 (32) | 5 (55·5) | 4 (44) |
| Normal | 0·001 | 25 | 21 (84) | 0 | 21 (100) |

* Percentage of mice with tumours calculated from total number of mice.
† Percentage of mice with regressed tumours calculated from number of mice developing tumours.
‡ Percentage of mice dead with tumours calculated from number of mice developing tumours.

TABLE II.—*Tumour Induction by MSV-M in BALB/c Mice Born to and Nursed by Variously Treated Mothers*[1]

| Nursing mother | Total no. mice | No. mice with tumours (%) | No. mice with regressing tumours (%) | No. mice dead with tumours (%) |
|---|---|---|---|---|
| MSV-M exposed[2] | 76 | 66 (87)* | 4 (6)† | 59 (89)‡ |
| Immunized *post partum* | 23 | 23 (100) | 0 | 23 (100) |
| Immunized before mating[3] | 60 | 22 (37) | 16 (73) | 8 (36) |

[1] Offspring were injected with 0·05 ml of 0·01 gEq MSV-M tumour extract.
[2] These mothers had previously nursed an MSV-M-injected litter.
[3] Time interval between immunization and parturition varied from 9 to 32 weeks.
* Percentage of mice with tumours calculated from total number of mice.
† Percentage of mice with regressed tumours calculated from number of mice developing tumours.
‡ Percentage of mice dead with tumours calculated from number of mice developing tumours.

Data obtained when 1–2 week old mice were nursed by variously treated mothers and injected with 0·01 gEq tumour extract are reported in Table II. A slight reduction in tumour incidence was observed in mice born to MSV-M exposed mothers (87% compared with 97·5% in controls). However, when tumour incidence was analysed in each single litter, only 3 out of 14 litters showed a decrease in incidence (Table III).

TABLE III.—*Tumourigenesis in MSV-M Injected BALB/c Mice Born to and Nursed by MSV-M Exposed Mothers*

| Litter size | No. mice with tumours | No. mice with regressed tumours | No. mice dead with tumours |
|---|---|---|---|
| 3 | 3 | 0 | 3 |
| >7 | 4 | 3 | 1 |
| 9 | 9 | 0 | 9 |
| 3 | 3 | 0 | 3 |
| 6 | 6 | 0 | 6 |
| >4 | 1 | 0 | 1 |
| 9 | 9 | 0 | 9 |
| 5 | 5 | 0 | 5 |
| 3 | 3 | 0 | 3 |
| 5 | 5 | 0 | 5 |
| 8 | 8 | 0 | 8 |
| >7 | 1 | 0 | 1 |
| 3 | 2 | 1 | 1 |
| 4 | 4 | 0 | 4 |
| 76 | 66 (87%) | 4 (16%) | 59 (89%) |

> Litters with reduced tumour incidence.

Mice born to mothers immunized *post partum* showed 100% tumours, in contrast with mice born to mothers immunized long before mating, which presented only 37% tumours, the majority of which eventually regressed. In the latter animals no differences were noted in tumour growth and regression per single litter with regard to the time interval between immunization and parturition. Even 32 weeks after immunization the mother could confer protection against MSV-M tumour development in her offspring (Table IV).

In order to obtain direct evidence for transfer of maternal immunity, the sera from mothers and their litters were tested for the presence of MSV-M neutralizing antibodies by means of a focus reduction method. From the results shown in Table V, it is clear that sera from immunized mothers and their offspring contained anti-MSV-M antibody (neutralizing titres of 8 and 4 respectively). In contrast, sera from normal mothers and their litters possessed no detectable neutralizing activity.

*Behaviour of induced tumours and rescue of MSV-M oncogenic activity*

Among the various experimental and control groups described above, no significant differences were observed in the behaviour of MSV-M induced tumours. The survival period of mice bearing progressive tumours was essentially the same, and the mice usually died within 1–3 weeks. Some tumours developed more than 2 months after MSV-M injection. Moreover, in some of the mice whose primary tumours had regressed 2–7 months earlier there was a reappearance of tumours, either at the injection site and/or

TABLE IV.—*Long-lasting Effect of Maternal Immunization on Tumour Induction by MSV-M in BALB/c Mice*

| Time interval between immunization and parturition (weeks) | Litter no. | Litter size | No. mice with tumours | No. mice with regressed tumours | No. mice dead with tumours |
|---|---|---|---|---|---|
| 9 | 13704 | 4 | 0 | 0 | 0 |
| 12 | 13750 | 3 | 3 | 3 | 0 |
| 13 | 13783 | 8 | 2 | 2 | 0 |
| 15 | 13816 | 5 | 4 | 3 | 1 |
| 11 | 13825 | 8 | 8 | 4 | 4 |
| 11 | 13835 | 9 | 3 | 2 | 1 |
| 11 | 13866 | 3 | 1 | 0 | 1 |
| 16 | 13926 | 6 | 0 | 0 | 0 |
| 19 | 14025 | 8 | 1 | 0 | 1 (late) |
| 32 | 14472 | 6 | 0 | 0 | 0 |
| | | 60 | 22 | 16 | 8 |

TABLE V.—*MSV-M Neutralization Titres of Sera from Immunized or Normal Mothers and their Offspring*

| Serum donors | Serum dilution | Average no. foci | Titre[3] |
|---|---|---|---|
| Immunized mothers[1] | 2 | 5 | 8 |
| | 4 | 3 | |
| | 8 | 18 | |
| | 16 | 29 | |
| Offspring from immunized mothers[2] | 2 | 10 | 4 |
| | 4 | 19 | |
| | 8 | 25 | |
| | 16 | 35 | |
| Normal mothers | 2 | 36 | <1 |
| | 4 | 36 | |
| | 8 | 39 | |
| | 16 | 38 | |
| Offspring from normal mothers[2] | 2 | 37 | <1 |
| | 4 | 38 | |
| | 8 | 38 | |
| | 16 | 38 | |

[1] BALB/c females received i.p. soon after mating 0·20 ml of gEq MSV-M, a second i.m. injection of MSV-M, at the same dose, was given 10 days later. Serum was collected and pooled 10 days after parturition.

[2] 9 mice, 10 days old, served as donors of pooled sera.

[3] Reciprocal of serum dilution that resulted in a 50% reduction of focus formation.

at different regions, *i.e.*, chest muscles, diaphragm and subcutaneous areas of the abdomen. Histologically, the tumours were essentially similar to those previously reported (Chieco-Bianchi *et al.*, 1971). The late appearing or reappearing tumours exhibited a marked lymphocytic infiltration within pleomorphic neoplastic cells, reminiscent of the morphological pattern of tumours undergoing regression.

Nine mice out of 642 inoculated with MSV-M at 7–14 days of age developed leukaemia at 5–12 months of age. Cell-free extracts were prepared from the lymph nodes, spleen and thymus of 6 out of these leukaemic mice. The extracts were diluted to 0·1 gEq and 0·05 ml injected i.m. into 1-week old mice. Table VI reports the donor history as well as the oncogenic activity of the leukaemic extracts thus prepared. Typical MSV-M tumours appeared 1–2 weeks after inoculation of 4 out of 6 of the extracts. Furthermore, extracts no. 2 and 5 gave rise to leukaemia in the recipient animals.

### DISCUSSION

Protection against leukaemia induction by Friend or Graffi virus has been observed in mice born to and nursed by specifically immunized mothers (Mirand, Grace and Buffet, 1966; Mathot and Scher, 1968; Chieco-Bianchi *et al.*, 1970); similar protection against Gross virus in rats has also been reported (Ioachim, 1970). The present study has revealed that tumourigenesis by MSV-M in BALB/c mice can also be remarkably reduced if the mother is specifically immunized. In addition, the immune state of the mother is enduring since MSV-M injected mice are protected against tumour development even when their mothers are immunized several months before conception. Not only did maternal immunity prevent

TABLE VI.—*Tumour Induction in Suckling Mice Injected with Extract from Mice that Developed Leukaemia after MSV-M Inoculation*

| Extract no. | Donor history: Mother | Donor history: Dilution of original MSV-M extract | Donor history: Tumour | Tumour induction by cell-free leukaemic extract[1] |
|---|---|---|---|---|
| 1 | Immunized | 0·01 | Regressed | 8/8 |
| 2 | Immunized | 0·01 | Late appearing | 2/3* |
| 3 | Immunized before mating | 0·01 | No | 0/4 |
| 4 | Normal | 0·01 | No | 9/11 |
| 5 | Normal | 0·01 | No | 0/5† |
| 6 | Normal | 0·001 | No | 1/7 |

[1] Number of mice with tumours/total number of mice.
* One mouse dead with leukaemia.
† Four mice dead with leukaemia.

tumour induction but, perhaps more significantly, it influenced the growth of established tumours, as shown by the occurrence of a good number of complete regressions in the various experimental groups. The protective phenomenon seen in the offspring of immune mothers is best explained by passive transfer (transplacental and/or by nursing) of humoral antibodies possessing neutralizing activity against MSV-M. Indeed, the results obtained by the *in vitro* focus reduction assay clearly demonstrate the presence of virus neutralizing antibodies in the sera of mice born to and nursed by immunized mothers. In consideration of a previous report (Bubenik *et al.*, 1969) suggesting that enhancement of tumour growth, rather than suppression, may result following the injection of anti-MSV-M immune serum, it is worthwhile to mention that such an effect has not been noted in the present experiments. It should be pointed out, however, that enhancement of tumour growth in the MSV-M and other tumour systems has been observed for transplanted rather than for developing primary tumours (Harvey and East, 1971; Kaliss, 1969).

Females immunized *post partum* did not transfer any protection from MSV-M tumourigenesis to their offspring. The fact that not enough time elapsed to develop an effective antibody titre after immunization might explain this failure. Also, it may be possible that protective antibody transfer occurs exclusively through the placenta during intrauterine life. This last possibility seems most unlikely, however, since passive transmission of maternal immunity in the mouse takes place mainly after birth through colostrum and milk (Brambell, 1970). Moreover, results of preliminary foster nursing studies performed in our laboratory indicate that mice born to normal mothers and nursed by immunized females do in fact show a lower incidence of MSV-M induced tumours (Chieco-Bianchi, unpublished results).

A reduced number of tumours was also observed in 3 out of 14 MSV-M-injected litters whose mothers had been previously exposed to the virus while nursing infected offspring. A similar observation has been made by Essex *et al.* (1971), who found that kittens born to and nursed by mothers that had previously nursed litters injected with feline leukaemia virus were protected from development of tumours following injection of feline sarcoma virus. These findings suggest that horizontal transmission of leukaemia and sarcoma viruses does occur, albeit rarely, and that it may have some practical significance under natural conditions as well (Brodey *et al.*, 1970).

A few MSV-M-injected mice did not develop tumours but subsequently developed leukaemia. While activation of " endogenous " virus cannot be completely ruled out, it is reasonable to assume that these leukaemias were induced by Moloney leukaemia virus which is usually present in high excess in MSV-M tumour extracts. However, why leukaemia should develop, even rarely, after MSV-M tumour regression is not clearly understood in view of the complete immunological cross reactivity observed between Moloney leukaemia and sarcoma viruses (Hartley and Rowe, 1966; Chuat *et al.*, 1969). It is possible that the immune system of the host is not operationally effective against leukaemia cells due to their lesser immunosensitivity or a " sneaking through " phenomenon. Another possibility is the fact that leukaemic cell kinetics follow a logarithmic growth curve which is unrelated to the viral supply. Development and growth of MSV-M tumours, on the other hand, seem to depend upon a constantly high rate of virus replication and continuous recruitment of newly infected transformed cells.

This last hypothesis, supported by *in vitro* (Bather, Leonard and Yang, 1968; Parkman, Levy and Ting, 1970) and *in vivo* findings (Chieco-Bianchi *et al.*, 1971) concurs with our present observation that passive immunity is capable of effecting tumour regression. Thus, when virus synthesis is slowed down by a host reaction or antibody transfer, prevention as well as tumour regression may result.

The recovery of MSV-M oncogenic activity from frankly leukaemic animals, of which some had never presented tumour, implies that MSV-M replicates at low levels during the host's life span. This is further substantiated by the late appearance or recurrence of tumours in some of the MSV-M inoculated mice. In fact, Blumenschein and Moloney (1969) have reported that virus is present in spleens from MSV-M injected mice even 46 days after inoculation, suggesting that the spleen and possibly other reticuloendothelial organs may be the carrier tissues for continuing infection in the animal. These considerations provide grounds for believing that when the host mechanisms constantly operating to limit oncogenic activity are undermined, then MSV-M may reach titres high enough to induce tumours.

Supported in part by grants from Consiglio Nazionale delle Ricerche and Associazione Italiana per la Promozione delle Ricerche sul Cancro.

### REFERENCES

BATHER, R., LEONARD, A. & YANG, J. (1968) Characteristics of the *in vitro* Assay of Murine Sarcoma Virus (Moloney) and Virus-infected Cells. *J. natn. Cancer Inst.*, **40**, 551.

BLUMENSCHEIN, G. R. & MOLONEY, J. B. (1969) Quantitative Dose-response Relationships of Murine Sarcoma Virus (Moloney) in BALB/c Mice. *J. natn. Cancer Inst.*, **42**, 123.

BRAMBELL, F.W.R. (1970) *The Transmission of Passive Immunity from Mother to Young.* Amsterdam: North-Holland Publishing Company.

BRODEY, R. S., MCDONOUGH, S. K., FRYE, F. L. & HARDY, W. D. (1970) Epidemiology of Feline Leukemia (Lymphosarcoma). In *Comparative Leukemia Research.* Ed. R. M. Dutcher. Basel: Karger. p. 333.

BUBENIK, J., TURANO, A. & FADDA, G. (1969) Prevention of Carcinogenesis by Murine Sarcoma Virus (Harvey) Following Injection of Immune Sera During the Latency Period. *Int. J. Cancer*, **4**, 648.

CHIECO-BIANCHI, L., FIORE-DONATI, L., COLLAVO, D. & TRIDENTE, G. (1970) Immunological Problems in Virus-induced Leukemia in the Mouse. In *Immunity and Tolerance in Oncogenesis.* Ed. L. Severi. Perugia: Division of Cancer Research. p. 599.

CHIECO-BIANCHI, L., PENNELLI, N., COLLAVO, D. & TRIDENTE, G. (1971) Tumor Induction by Murine Leukemia/Sarcoma Viruses: Morphological and Immunological Studies. In *Morphological and Functional Aspects of Immunity.* Ed. Lindahl-Kiessling, Alm and Hanna. New York: Plenum Publishing Corp. p. 555.

CHUAT, J. C., BERMAN, L., GUNVEN, P. & KLEIN, E. (1969) Studies on Murine Sarcoma Virus: Antigenic Characterization of Murine Sarcoma Virus Induced Tumor Cells. *Int. J. Cancer*, **4**, 465.

ESSEX, M., KLEIN, G., SNYDER, S. P. & HARROLD, J. B. (1971) Antibody to Feline Oncornavirus-associated Cell Membrane Antigen in Neonatal Cats. *Int. J. Cancer*, **8**, 384.

FEFER, A., MCCOY, J. L. & GLYNN, J. P. (1967*a*) Antigenicity of a Virus-induced Murine Sarcoma (Moloney). *Cancer Res.*, **27**, 962.

FEFER, A., MCCOY, J. L. & GLYNN, J. P. (1967*b*) Induction and Regression of Primary Moloney Sarcoma Virus-induced Tumors in Mice. *Cancer Res.*, **27**, 1626.

FEFER, A., MCCOY, J. L., PERK, K. & GLYNN, J. P. (1968) Immunologic, Virologic and Pathologic Studies of Regression of Autochthonous Moloney Sarcoma Virus-induced Tumors in Mice. *Cancer Res.*, **28**, 1577.

HARTLEY, J. W. & ROWE, W. P. (1966) Production of Altered Cell Foci in Tissue Culture by Defective Moloney Sarcoma Virus Particles. *Proc. natn. Acad. Sci. U.S.A.*, **55**, 780.

HARVEY, J. J. & EAST, J. (1971) The Murine Sarcoma Virus (MSV). *Int. Rev. exp. Path.*, **10**, 265.

HOOK, W. A., CHIRIGOS, M. A. & CHAN, S. P. (1969) Increased Tumorigenesis of Murine Sarcoma Virus (Moloney) by Co-infection with Rauscher Virus or by Treatment with Antilymphocyte Serum. *Cancer Res.*, **29**, 1008.

IOACHIM, H. L. (1970) Prevention of Gross Virus-induced Leukemia in Progeny Immunized Female Rats. *Cancer Res.*, **30**, 2661.

KALISS, N. (1969) Immunological Enhancement. *Int. Rev. exp. Path.*, **8**, 241.

LAW, L. W., TING, R. C. & ALLISON, A. C. (1968) Effects of Antilymphocyte Serum on Tumour and Leukaemia Induction by Murine Sarcoma Virus (MSV). *Nature, Lond.*, **220**, 611.

MATHOT, C. & SCHER, S. (1968) Transmission through Milk of Antibodies against the Friend Virus in Mice. *Nature, Lond.*, **219**, 82.

MIRAND, E. A., GRACE, J. T. JR. & BUFFET, R. F. (1966) Passive and Active Immunity to Friend Virus Disease. *Nature, Lond.*, **209**, 696.

PARKMAN, R., LEVY, J. A. & TING, R. C. (1970) Murine Sarcoma Virus: the Question of Defectiveness. *Science, N.Y.*, **168**, 387.

PEARSON, G. R., REDMON, L. W. & BASS, L. R. (1973) Protective Effect of Immune Sera Against Transplantable Moloney Virus-induced Sarcoma and Lymphoma. *Cancer Res.*, **33**, 171.

SHACHAT, D. A., FEFER, A. & MOLONEY, J. B. (1968) Effect of Cortisone on Oncogenesis by Murine Sarcoma Virus (Moloney). *Cancer Res.*, **28**, 517.